# A Long and Winding Road: Dementia Caregiving With Grit and Grace

**DOI:** 10.1093/geroni/igz021

**Published:** 2019-08-23

**Authors:** Karen A Roberto, Brandy Renee Mccann, Rosemary Blieszner, Jyoti Savla

**Affiliations:** 1 Institute for Society, Culture and Environment, Virginia Tech, Blacksburg, VA; 2 Center for Gerontology, Virginia Tech, Blacksburg, VA; 3 Departments of Internal Medicine and Psychiatry and Behavioral Medicine, Virginia Tech Carilion School of Medicine, Roanoke, VA; 4 Department of Human Development and Family Science, Virginia Tech Blacksburg, VA

**Keywords:** Dementia, Family caregivers, Longitudinal design, Qualitative analysis

## Abstract

**Background and Objectives:**

Many dementia caregivers provide care for numerous years. Exhibiting grit, or commitment and persistence in the face of adversity, may bolster their ability to manage caregiving challenges. We explored grit in relationship to memory and behavior problems and response to stressors among women engaged in long-term dementia care.

**Research Design and Methods:**

Informed by a life course perspective, and guided by stress-process theory, we interviewed 10 women with a spouse or parent initially diagnosed with mild cognitive impairment 4 times over 10 years. Using Charmaz’s analysis methods and grit as a sensitizing concept, we employed an unfolding analytic strategy involving (a) thematic analysis to identify expressions of grit in response to caregiving stressors across interviews and (b) case-by-case comparisons to assess associations of grit with the use of care strategies across caregivers over time.

**Results:**

Dementia caregivers experienced unrelenting and changing psychosocial and physical challenges. Over time, most women exhibited a sustained commitment to the relationship through the ways in which they protected the identity of the person with dementia, modified their expectations for emotional intimacy, and managed their financial affairs. They persevered as their roles and relationships fluctuated, often finding purpose and relief through employment and leisure pursuits. As care intensified, women who took charge and consciously made decisions in the best interest of the care recipient and themselves minimized stress.

**Discussion and Implications:**

While some caregivers exhibited grit from the outset, all showed enhanced perseverance and commitment to the ways they managed memory-related changes over time. Developing confidence in their ability to manage and provide care helped the caregivers respond to stressors with purpose and sustain their roles and responsibilities. Enhancing grit in long-term dementia caregivers may result in better individual and relational outcomes.

**Translational Significance:**

Caregivers were committed to and took charge of their situations as they made decisions in the best interest of themselves and their relative with memory impairments. Findings provide a basis for designing interventions to enhance grit levels and engage caregivers in the process of learning and relearning ways of adjusting psychologically and socially to changes in their roles, responsibilities, and relationships.

More than 15 million families provide informal care for people with Alzheimer’s disease and other dementias (PwD; Alzheimer’s Association, 2018). More than half of them are wives and adult daughters who provide care for 4 years or longer. A diagnosis of mild cognitive impairment (MCI) often signals a transition into caregiving, as older adults with MCI begin needing help with social and behavioral tasks, such as remembering appointments and balancing their checkbooks. If they experience further decline, their need for care intensifies, requiring caregivers to manage the provision of care and most often, directly assist with daily activities and personal care (Kasper, Freedman, Spillman, & Wolff, 2015; Riffin, Van Ness, Wolff, & Fried, 2017).

Most studies on adjustment to caregiving have taken a retrospective snapshot at one point in time (O’Connor, 2007) or examined short-term effects of an intervention (e.g., Gaugler, Reese, Mittelman, 2018; Montgomery, Kwak, Kosloski, & Valuch, 2011). Researchers typically do not control for length of dementia-related caregiving or examine caregivers’ experiences and ways of coping as their relatives’ cognitive abilities decline. Because caregiving is not a static process and longitudinal research on caring for PwD is sparse, we launched a prospective caregiving study of families in which one member initially diagnosed with MCI experienced progressive memory decline over time (Roberto, Blieszner, McCann, & McPherson, 2011). We extended the original study through four waves of data collection over a decade. For the current analysis, we focused on 10 women who served as the primary caregiver for approximately 10 years to a relative who progressed from MCI to more advanced dementia during the study to explore areas of stress and concern. We explored how these long-term caregivers sustained their commitment and exerted perseverance during their caregiving career.

## Theoretical Frameworks

This investigation relied on the life course perspective (Elder, 1998), Pearlin’s stress-process model (Pearlin, Mullan, Semple, & Skaff, 1990), and notions of grit (Duckworth, 2016) to inform understanding of the care trajectories of long-term dementia caregivers. The overarching life course perspective draws attention to and integrates life pathways, relationships, and social and cultural factors, enabling us to connect needs of the PwD with the caregiver’s abilities, beliefs, roles, and responsibilities (Elder, 1998). According to this framework, people experience sequences of transitions and periods of stability throughout life that form distinctive trajectories in various life domains (Elder, 1998). For example, assuming care for an older family member is a transition signaling a major life change. From that point forward, an individual is no longer only a spouse or child, but also a caregiver who will likely experience numerous care-related transitions over the course of the care recipient’s illness. Further, this perspective posits the principle of linked lives, which emphasizes relational interconnectedness (Elder, 1998). Individual lives are embedded within and influenced by relationships with family and other social network members. The ways in which caregivers manage changes in their care responsibilities are often contingent upon, and sometimes conflict with, behaviors and beliefs of the care recipient as well as other family members.

Pearlin and colleagues’ (1990) caregiving stress framework highlights the interplay of four sets of variables that affect outcomes of difficult situations: background and context variables, such as the caregiver’s relationship with the care receiver and resources for dealing with the situation; primary stressors, including the care receiver’s cognitive losses and problematic behaviors; secondary stressors such as the caregiver’s experience of role and relationship strains beyond those of caregiving and the caregiver’s sense of self-efficacy and related psychological states; and enabling factors such as coping resources and support from others. These enabling factors may exacerbate or ameliorate primary and secondary stressors and outcomes.

While the concept of grit (Duckworth, 2016), a person’s capacity to remain intensely passionate about and persistent in pursuing goals over time, has been used to study why some individuals are successful in achieving their educational, professional, and creative goals (Duckworth, 2016; Eskreis-Winkler, Duckwork, Shulman, & Beal, 2014), it is also useful for understanding long-term caregiving. Passion refers to a person’s commitment (i.e., enduring interest or devotion; purpose) to pursuits and long-term goals; perseverance (i.e., tenacity, doggedness; stamina) refers to a person’s ability to sustain hard work over long stretches of time in the face of obstacles and adversity. In this article, we use the term “commitment” rather than “passion” because few people are passionate about caregiving activities. Nevertheless, caregivers often show deep devotion while caregiving and feel a strong sense of purpose and duty towards their relatives needing care. Thus, we conceived of “grit” in terms of the women’s commitment to the family caregiver role, their willingness to embrace the work of caregiving, and their perseverance in finding ways to sustain this role over time. Because few longitudinal dementia caregiving studies currently exist, these theoretical features of grit have yet to be examined among family caregivers.

Some researchers may argue that grit is similar to coping and resilience conceptually. Common among the many ways of coping and definitions of resilience is a positive response to adversity. While Duckworth has noted that part of what it means to be gritty is to be resilient (Perkins-Gough, 2013), she argues that having focused passion and deep commitment over a long-term period is what distinguishes grit from resilience. Thus, grit in the context of caregiving represents caregivers’ commitment and perseverance, which prevents them from abandoning their responsibilities and allows them to find ways to endure and develop a sense of joy and purpose in the caregiving role. Similar to findings regarding resilience among caregivers (Dias et al., 2015), there are likely to be multiple biopsychosocial factors that interact and are associated with grit in dementia care, including contextual, care recipient status, and resource indicators (Gaugler, Kane, & Newcomer, 2007). Because long-term caregiving is not a single event, but rather a complicated sequence of events that occur over time, the concept of grit, as used in this study, captures caregivers’ unfolding determination to provide care to a loved one, despite the continual stress they face. Thus, while grit and resilience processes share a focus on the importance of perseverance, grit also considers of equal importance the caregivers’ commitment and purpose. It has the potential to deepen understanding of the negative strains along with positive benefits of long-term caregiving (Hill, Burrow, & Bronk, 2016).

Duckworth (2016) pointed to the importance of identity, or how people see themselves, as foundational to having both commitment (i.e., purpose) and perseverance. Family caregivers have a preexisting relationship with PwD that typically involves a mutual exchange of help, support, and intimacy. When they find themselves caring for a loved one with dementia, they usually have a period of identity adjustment as they redefine their relationship. Although the experiences of caregivers are diverse, Montgomery and Kosloski (2013) identified two fundamental aspects to the process of becoming a caregiver. First is the help the caregiver provides (e.g., protecting feelings, managing medications, making lists), and second is how family members make sense of those activities. For example, some caregivers may see writing reminders for PwDs as an expected extension of their spousal role, whereas others may interpret making notes as signaling relational loss. The period of identity adjustment is likely to vary depending on PwDs’ symptoms and caregivers’ ability to reevaluate their sense of purpose in the relationship.

### Dementia Caregiving

#### Changes in Cognitive Abilities

MCI is an early state of cognitive decline when noticeable changes in memory or cognition occur, but the changes are believed not to interfere significantly with everyday functioning (Petersen, 2004). Intermittent forgetfulness, as well as minor behavioral changes and emotional outbursts, often are the first signs of MCI. Caregivers of elders with MCI may feel stress and strain in coping with such changes (Bruce, McQuiggan, Williams, Westervelt, & Tremont, 2008; Paradise et al., 2015; Roberto et al., 2011). Although symptoms may last for years and the prognosis is uncertain (Alzheimer’s Association, 2018; Petersen, 2004), most persons with MCI will progress to more severe dementia (Petersen et al., 2001). With further cognitive decline, new or more demanding behavioral problems emerge, and the time commitment and intensity of needed care increases.

Although family caregivers report positive feelings about caregiving (Lloyd, Patterson, & Muers, 2016), they also report high levels of stress, anxiety, and depression (Pearlin et al., 1990). Subtle changes in the relationship, as well as decline in cognitive functioning, may be especially burdensome. How caregivers cope with cognitive and relational challenges over time may depend on their individual resilience and commitment to the relationship (Dias et al., 2015).

### Long-term Commitment to Caregiving

Many people expect to give care to their spouse or parents in late life and feel a strong sense of commitment to their family members (Montgomery & Kosloski, 2013). Yet, the unexpected loss of emotional closeness can be a challenging transition, especially early in the disease trajectory when the person with MCI is otherwise functioning as usual. Caregivers of PwD reported gradual loss of an “everyday sense of intimacy” (Youell, Callagan, & Buchanan, 2016) as they were able to do fewer and fewer things with their partner such as conversing and participating in shared activities (e.g., shopping; hobbies) or social gatherings (e.g., time with friends; community events). Additionally, caregivers may not feel disposed towards emotional or physical intimacy with the PwD who has lost interest in the life they once shared.

One of the ways that caregivers show their sustained commitment to their loved one is to help the PwD adjust to cognitive decline (Hayes, Boylstein, & Zimmerman, 2009; MacRae, 2011). Wawrziczny, Antoine, Ducharme, Kergoat, and Pasquier (2016) interviewed 16 spouse dyads to examine how MCI affected intimate relationships. The caregivers engaged in protective behaviors, typically guarding the feelings of PwD. They diverged, however, in how they assessed the need for help because many PwD resisted accepting help or even recognizing that they might need help, which was stressful for the caregiver. Because those with MCI typically manifest day-to-day variation in cognitive functioning, it became difficult to predict when help was needed, and caregivers had to stay vigilant. They reported retrospectively that as the symptoms progressed over time, the need to take charge of household matters (e.g., doing most chores) became more important than protecting the PwD’s feelings. This change in priorities led most of the caregivers to feel that intimate aspects of their relationships were over, although their commitment to sustaining the relationship remained strong (Wawrziczny et al., 2016).

Adult children face similar relational challenges when caring for their parents. Trying to prevent their parents from experiencing distress from being forgetful, adult daughters often expressed uncertainty about how to respond to specific situations and doubt about their ability to provide care without upsetting their parents (Day, Anderson, & Davis, 2014). Even though their relationship had changed, the daughters reported a close emotional attachment to their parents that helped sustain their motivation to provide care. At the same time, the researchers speculated that the daughters’ inability to detach from the caregiving situation may ultimately lead to feelings of helplessness, hopelessness, and social isolation that could reduce their satisfaction with the relationship and effectiveness as caregivers.

### Caregiver Identity and Meaning-Making

Caregivers commonly report that their identity as a caregiver becomes salient only when caregiving duties take precedence over other roles and the relationship with the care recipient clearly changes (Eifert, Adams, Dudley, & Perko, 2015). This meaning-making process may take years. O’Conner (2007) found three areas of tension during this process: (a) refocusing, so caregivers paid more attention to self-care while coping with feelings of guilt; (b) disentangling themselves from preexisting emotional bonds—almost a process of objectification of the elder needing care; and (c) rebalancing relational power dynamics when having to assume management of the other persons’ life. Although these areas of tension and meaning-making can be challenging, they may help caregivers’ access and strengthen their commitment and perseverance over time as they learn to identify what problems they can solve, such as employing self-care, and what situations they must accept and redefine as permanent changes in the relationship (Chen, 2016).

## Method

### Sample

The women in the current study participated in a larger investigation of dementia caregiving which began in 2004 when a relative was diagnosed with MCI at a memory clinic (see Roberto et al., 2011 review for method details). The purpose of the original study was to gather information on family members’ initial caregiving experiences for a relative with MCI and explore how these experiences changed over time. The first wave included 125 family members identified as primary care partners for persons with MCI. The time between MCI diagnosis and the initial interview (T1) ranged from 0.5 to 46 months (*M* = 10.01 ± 10.69 months). Eighty-five care partners were interviewed in the second wave (T2 averaged 13.04 ± 2.35 months from T1) and 52 care partners participated in the third wave (T3 averaged 35.56 ± 8.98 months from T2). The final wave of data (T4) was collected from 28 caregivers approximately 10 years after T1. Primary reasons for caregiver attrition across the four waves of data collection were caregivers’ disinterest in continuing in the study (*n* = 30), death of either the PwD or caregiver (*n* = 24), and lost to follow-up (*n* = 22).

For the current analysis, we selected primary caregivers who had participated in all four waves of data collection and were still providing in-home care for PwD (T1–T4; *n* = 10; see Table 1). At T1, these caregivers, all women, were 28 to 72 years old. Eight were wives caring for their husbands to whom they had been married for 1 to 50 years. One caregiver was a daughter, and another was a granddaughter; both cared for women who co-resided with them. Using the date of the initial MCI assessment as a proxy indicator for the first year of caregiving, these 10 participants began their care journey 9.1 years (±1.05; range 6.8–10.0) prior to the T4 interview.

**Table 1.** Demographic Characteristics of Caregivers and Persons with Dementia

Caregivers					Persons with dementia			
Pseudonym	Race	Age, T4	Relationship to PwD	Years married, T1	Sex	Race	Age, T4	Months since Dx, T1
Helen	White	80	Spouse	48	M	White	87	12
Wendy	White	74	Spouse	47	M	Other	75	22
Gail	White	61	Adult child	–	F	White	85	8
Delia	African American	69	Spouse	20	M	African American	75	2
Hilda	White	80	Spouse	45	M	White	81	13
Krystal	White	77	Spouse	50	M	White	78	<1
Nadine	White	36	Grand-daughter	–	F	White	76	<1
Tracy	White	80	Spouse	25	M	White	69	38
Shirley	White	72	Spouse	1	M	White	85	1
Bertha	White	73	Spouse	3	M	White	82	12
*M*		70.3		29.8			79.3	

*Note*. PwD = Person with Dementia; Dx = Diagnosis.

The PwD ranged from 61 to 76 years of age at TI and had a clinical diagnosis of MCI (see Roberto et al., 2011 for details). We did not reassess cognitive function in subsequent waves because interviews were conducted at participant’s homes or on the phone. However, we confirmed the diagnosis of dementia with family caregivers at each wave before follow-up interviews were conducted.

### Procedure

In-person interviews were conducted by research team members at T1, T2, and T3. The interviews included a series of measures commonly used in caregiving studies and 12 open-ended questions about the PwD’s memory loss and other health conditions and the caregiver’s roles, responsibilities, and relationships. Given the dearth of research on MCI caregivers at TI, we explored caregivers’ perceptions of PwD’s memory loss symptoms and reactions, as well as details about their roles, responsibilities, and relationships as they began the caregiving journey (Supplementary Appendix). The questions and their probes were modified for subsequent waves of data collection to reflect what was previously learned and to capture changes in the caregiving experience. The open-ended portion of the interviews was tape-recorded and transcribed verbatim. The interview data for the 10 cases in this study resulted in 491 transcript pages. Interviews at T4 were conducted by telephone. Caregivers responded to questions about their current care situation and caregiving measures asked in previous waves (e.g., memory problems and behaviors; burden).

The Institutional Review Boards of Virginia Tech and the participating memory clinics approved this research. Pseudonyms were assigned to each caregiver and PwD to ensure confidentiality.

### Interview Data Analysis

To analyze the qualitative data, we used Charmaz’s (2006) methods for grounded theory as a guide. Specifically, we used open coding and focused coding as the two overarching phases of data analysis.

#### Open coding

T1 to T3 interview data were coded and managed using Atlas.ti software. The research team included multiple coders who met regularly to establish a coding scheme, achieve consensus on the meaning of coding categories, and develop a protocol to manage adding or combining codes (Roberto et al., 2011). These collaborative coding sessions fostered development of a shared, detailed understanding of the rich interview data. Team members organized the codes into general categories using sensitizing concepts from Pearlin and colleague’s (1990) Stress Process framework and other caregiving literature. Examples of coding categories (and codes) are Everyday Life (symptoms, others’ awareness), Problems (behaviors, emotions), Physical Health (PwD, caregiver), and Social Support (preferences, use/non-use, relationships).

#### Focused coding

For the current analysis, we employed an unfolding analytic strategy to identify and develop findings relevant to questions about long-term dementia caregiving. First, we conducted a basic thematic analysis using the open coding categories, where evidence of grittiness began to emerge, and identified new themes particular to the 10 primary caregivers who participated in all four study waves. Next, we completed case-by-case comparisons to assess the ways in which themes were nuanced across caregivers and identified areas of congruence and difference. Specifically, we examined passages under particular codes and compared quotes to each other in the context of the caregivers’ overall stories. We then focused attention on how themes changed across time for each participant. Throughout this process, we kept theoretical and analytic memos related to the life course perspective (Elder, 1998), stress and coping (Pearlin et al., 1990), and the concept of grit (Duckworth, 2016), revising and refining throughout the process.

Using the Coping category to illustrate our analytic process, we noted that seven women mentioned their career as a source of relief for the stress of caregiving; it gave them a sense of purpose outside of caregiving. We identified the women who talked about work and the ones who did not. For women who did not specifically mention paid work, we reexamined their interviews to see what meaningful activities, if any, might be similar to paid work. Then we considered how all the women talked about work and other meaningful activities and determined whether they helped the caregivers to access and strengthen their grit as they transitioned into caregiving. Finally, we examined how this coping resource changed over time in the context of their caregiving journeys.

### Findings

Overall, caregivers exhibited commitment, purpose and perseverance, or grit, as they adjusted to changes in their caregiving roles, responsibilities, and relationships. At first the PwD’s MCI was recognized by the caregivers as small memory and personality changes that did not present major obstacles to the PwD’s daily functioning nor create much stress in the caregivers’ lives, allowing the women to be gritty about maintaining the status quo of their relationships and the PwD’s identity in social settings.

As changes intensified, however, two domains were frequently mentioned as sources of stress, likely because they were difficult to manage: emotional intimacy in the relationship and financial affairs. Although these aspects of relational change made caregiving stressful, over time, the women adjusted to the changes by adopting a caregiving role, persevering, and remaining committed to the relationship. Two ways the women persevered in their role and found relief from early caregiving strain were through their participation in paid employment and serious leisure activity (i.e., a rewarding substantial commitment or systematic pursuit that results in a sense of mastery; Stebbins, 2015). Both strategies helped deepen the caregivers’ identity and sense of purpose outside the relationship with PwD while sustaining their commitment to providing care and support.

In presenting the findings below, we provide examples of how the caregivers experienced changes over time and exhibited grit. It is important to note that the caregivers’ grit is on a continuum and some women likely began the study more gritty than others.

### From Managing PwD’s Identity to Becoming a Caregiver

At T1, the women discussed guarding the PwD’s identity both in the community and within their homes. Their purpose was to protect the PwD from negative appraisals by others in the community and to keep them engaged in meaningful activities in an effort to slow cognitive decline. As time and symptoms progressed. However, the women’s ways of showing commitment in the relationships changed from managing social situations and household tasks to providing more instrumental care.

Typically, the women accompanied the PwD in social situations and acted as a personal memory aid. For example, Gail said her mother, Gay, was nervous about social interactions; over time, she had become increasingly dependent on Gail’s intervention when interacting with others. By T2, Gay wanted Gail to accompany her at friend gatherings because she depended on Gail to prompt her memory. At T3, however, Gail was no longer able to protect Gay’s social identity; her purpose had changed to protect her feelings. Gay had become more withdrawn as her memory problems worsened and the family depended on a home health worker during the days when Gail was at work. Gail said she wished her mom were more independent but does not mention that to Gay because she does not “want to make her feel worse about [her memory loss].” Although Gay’s memory problems had plateaued by T4, she had experienced declining physical health. Gail was adjusting to these new caregiving duties, but she expressed uncertainty about her ability to care for Gay and manage the other demands on her time. Clearly committed to the relationship, but stressed, Gail’s perseverance to continue to provide care appeared to be waning.

Similarly, at the beginning of her caregiving journey, Bertha said, “you just try to anticipate what could go wrong and try to head it off,” such as discouraging the children and grandchildren from visiting because being around their relatives “rattled” him. Further, at T1 Bertha and her husband Buddy were active in their church, but other congregants did not know about Buddy’s memory problems. She downplayed his health problems with other church members (such as when he needed to use a cane temporarily), saying “[Buddy] doesn’t like anybody to know that he’s sick. That’s a big sign of weakness or something.” By T2, they were unable to hide Buddy’s memory problems when he forgot to pick up other church members for an outing. Bertha reported they “took it in stride.” Because his memory problems were becoming undeniable, protecting his identity became less important and Bertha’s purpose changed. By T3 and T4, Buddy’s declining physical health and strained family relationships were their biggest problems, with Bertha saying they no longer attended church on a regular basis because Buddy had a hard time walking from the car to the building. He’d had a minor stroke that had further affected his memory, but, for her, the biggest issue was changes in his mood. He was prone to angry outbursts that caused issues with other family members, particularly their grandson who often stayed with them. Thus, over time, Bertha was doing more caregiving work as Buddy’s physical and mental health declined and talked less about their relationship.

Church was a worrisome arena for Hilda and her husband Harlan too, but Hilda developed strategies to help Harlan feel independent. She provided several examples of efforts she made so Harlan would not feel bad about his memory problems. At T1, Hilda said her strategies were to “leave things [the way they used to be] around the house” and to continue with social outings and actively participate in their church choirs. At T3, both Hilda and Harlan still participated in the choir although Harlan’s cognitive problems had worsened. He needed assistance, such as keeping up with page numbers, and Hilda found herself always watching out for him so he did not get too frustrated or upset. She revealed that both at home and in social settings, she tried to do as much together as possible to keep Harlan on task. By T4, Hilda reported Harlan’s memory and behavior problems had worsened, suggesting she had lost a sense of purpose in her ability to manage his behavior. She said that she felt uncertain about their future. Although we do not know for how long Hilda continued as Harlan’s primary caregiver, it was clear that she was at a juncture that challenged her ability to be gritty.

The caregivers also discussed how they handled memory loss in their everyday interactions around the house. Delia is representative of other wife caregivers in discussing how she protected her husband Darnell’s feelings and allowed him some illusion of control, saying at T1, “I have to be able to change along with him.” In the beginning, she often compared Darnell to her mom, who had dementia. As her mother’s primary caregiver, Delia found her mom willing to accept help from others; however, the situation with Darnell was more difficult. His symptoms were less pronounced, and he had always been independent in their relationship. At T1, she said she had to be “reprogrammed” in adjusting to the fact that Darnell was not dependable to take phone calls or run errands, but said she was not ready to reduce his independence yet. Delia’s response reflected grit as she was committed to helping Darnell preserve his autonomy and identity in the face of his memory problems. Communication and interaction between Delia and Darnell had become strained by T2; they were avoiding each other, suggesting that Delia was at another juncture in reevaluating her sense of purpose. Delia sought marriage counseling, learned more about dementia, and decided to forego telling Darnell of his memory mistakes. At T3, she reported Darnell had become withdrawn. Delia showed her perseverance by looking for ways to help him participate in household management that did not depend on his memory, such as walking the dogs or cleaning and rearranging the furniture. By T4 Delia seemed less concerned about her relationship with Darnell, reporting that her own health problems and increasing stress in the extended family were making it difficult to care for Darnell. Like Hilda, Delia was at a point in her caregiving career where other personal and relationship challenges made it difficult for her to sustain her grittiness.

For another couple, Kent’s memory problems remained stable over time whereas Krystal’s physical health deteriorated. She characterized their relationship as one of interdependence and saw the emergence of their memory and health problems, respectively, as another way in which they had to depend on each other. At T1, Krystal felt challenged because she wanted to bring to Kent’s attention to things she felt he should remember, such as turning off a faucet. By T3 she was showing more grit as she had become more accepting of Kent’s memory issues, telling their adult children to contact her directly if they needed something because Kent would not remember to give her messages. She only brought up issues or concerns with him if they were truly important, such as driving. She “didn’t want to make him feel bad,” but thought he should try harder to overcome his limitations. Krystal said Kent had become worse at driving (more aggressive) and at the same time was more sensitive to criticism. She felt challenged in discussing driving issues with him, saying, “I think it probably makes him feel diminished or something, […] but at the same time I do not want him to hurt anyone.” By T4 Krystal’s major concern was providing support to Kent as his physical health had steadily declined. With each progression of Kent’s decline, Krystal persevered, adjusting to the changes and remaining committed to the relationship.

Although the caregivers found ways to help their PwD maintain independence for as long as possible in social settings, two other areas challenged their ability to persevere throughout the course of dementia: decreased emotional intimacy and managing financial affairs. Both domains pertain to home life, suggesting that finding ways to stay committed and persevere may be more difficult in the context of interpersonal relationship dynamics.

### From Decreased Emotional Intimacy to Prioritizing Self-Care

As caregivers become aware that they are doing more and more for the PwD, the relationship suffers in terms of emotional intimacy (Eifert et al., 2015). These changes force caregivers to reassess their roles, which was often a long process. A large part of transitioning into a caregiver identity was a reinvestment from an emotional relationship with the PwD to finding more meaning in other activities. For example, at T1 Wendy said that she and Walter still shared conversation and travel but that she had begun to remove everyday irritations, such as not using the computer when Walter was around because he got upset when she did. At T2 and T3, Wendy continued avoiding activities and conversations that upset Walter. For example, their extended family had chronic issues about which Wendy had been able to vent to Walter before, but as his cognitive problems worsened, she could no longer discuss relatives for fear of sending him into a downward mood spiral. At T3, she indicated she could no longer share concerns with him and missed the emotional support they once shared. Although the extended family had become more supportive as they became aware of Walter’s cognitive problems, which reduced an external stressor and provided emotional relief for Wendy, they provided little, if any, actual assistance or help with Walter’s care. Wendy was having trouble keeping up with the care of their home and animals, which previously had been outlets for her, but found ways to continue in her part-time work (self-employed), such as taking Walter with her when she needed to run errands. At T4, Wendy reported Walter’s memory problems had increased, and she perceived his care as more burdensome.

As was common among the caregivers, Helen described gradual erosion of intimacy over time, saying it took her a while to acknowledge such changes in Harold because he had always been reticent. At T1, she was frustrated with his memory problems and his visits to the doctor without her, preventing her from knowing what was going on. By T3, Helen had stopped all physical intimacy with Harold, and their emotional intimacy had also declined. She said Harold was very argumentative and she avoided conflict by keeping concerns to herself. Both Helen and Wendy were saddened by the loss of spousal emotional intimacy and struggled with adjusting expectations of their husbands’ ability to be emotionally engaged. However, with increasing symptoms on Harold’s part, Helen had developed her caregiver identity more fully and lowered her expectations for an emotional relationship with Harold. She had held on to a part-time position at her job that afforded her the opportunity to develop purpose in emotionally fulfilling relationships with co-workers and friends.

Not surprisingly, adult children caregivers also had to make emotional adjustments as the PwD changed over time. Although these two relationships differed from other dyads in the type of intimacy and gender dynamics (i.e., spousal vs. parental), both relationships were marked in their emotional and physical closeness. The younger women in the dyads spoke of a deep friendship born of close physical proximity with the PwD.

At first, Gail still felt a sense of closeness with her mother Gay because they engaged in the same hobbies together. A favorite pastime was gardening when Gail returned from work. But by T3, Gay no longer wanted to garden. Showing her commitment and compassion (i.e., grit), Gail said she was gradually accepting her mother’s changes: “I have learned through my life that nothing is permanent. […] This is just where we are right now. […] So what if you have to [repeat yourself] eight times.” Although mother and daughter were very close emotionally, it is notable that Gail was married, thus having another close relationship in which to invest her emotional self. In contrast, Nadine hoped that “even if [momma] gets Alzheimer’s, we will still be close [and] she’ll learn to rely on me.” Although the desire for ongoing closeness suggests that Nadine was still invested in an emotional relationship with her mother, she kept mentioning that Nellie had taken her in as a child and become her mother, so she wanted to return the favor now that Nellie was vulnerable. In the later interviews, she said that her job was her “outlet” but that she had rearranged her work schedule so as to provide more care as Nellie’s symptoms worsened.

As noted earlier, only one pattern emerged from questions about what these women did for themselves to deal with the stress of caring for a PwD. Across time, all of them named paid work or serious leisure pursuits (Stebbins, 2015), such as caring for animals, as their primary source of self-care and enjoyment. They displayed a growth mindset and found ways to maintain these activities outside the relationship with the PwD. For example, in addition to traveling and visiting church family, Tracy enjoyed keeping busy by maintaining a small business the couple owned. Similarly, Krystal said the only place she got “rest” was when she was at work driving a school bus; and Nadine referred to her nursing job as her “release.” Wendy, Shirley, and Bertha named household work and serious leisure pursuits as their “outlets” for dealing with the strains of PwD caring. Wendy, Nadine, and Bertha all cared for multiple animals, and Shirley sewed and spent time with others who enjoyed that hobby. That many of these women engaged in work involving care provision in some form reiterates that the relational aspects of caring for an emotionally close relative were particularly stressful, but the women still found meaning and a sense of purpose in providing care.

### From Financially Vulnerable To Fiscally Stable

One area of concern for eight of the 10 caregivers was financial management. Three wives set up separate checking accounts to ensure that household expenses were covered. The other three also worried about finances because they, too, observed that PwD was no longer able to manage money. By T3, both daughters had transitioned into handling their mothers’ financial affairs. They reported no conflict in the transition, as the PwD recognized that they were no longer able to manage money on their own.

Several of the spouse caregivers experienced conflict over finances as they attempted to persevere in their caregiving role by taking control of the family money. This is an area where it may be especially difficult to show grit, particularly in relationships where husbands held power over money management. As previously noted, Delia reported she and Darnell had always had a rocky relationship, and he was especially independent. At T1, she was already experiencing conflicts over money: “When you have a problem in your marriage, then you have a spouse that is now sick, with a memory problem, then you have a compound problem. We have a problem with him spending and buying.” She continued to report this concern at T2, saying they got separate checking accounts to deal with his spending, although this did not relieve her worry about Darnell overdrawing his account. Problems with finances were compounded by Darnell’s becoming extremely argumentative. Delia said, “I have to be very careful in how I approach him” with any concern, such as overspending, or eating foods that aggravated his diabetes, or driving aggressively. Like others, she wanted to protect his sense of self and confidence in his ability to handle everyday matters, including the finances. By T3, Darnell had become more accepting of his limitations, allowing Delia to take responsibility for their finances and management of their everyday lives more easily. At T4, Delia reported a notable decline in how troubled she was by Darnell’s memory problems; she was less worried about his spending and more concerned about keeping him engaged. Thus, over the course of a decade, Delia had learned to manage challenging caregiving tasks such as financial management and developed a sense of purpose around these caregiving activities.

Shirley was also struggling with financial issues at T1, saying that Scott was “in bad shape at the bank” after they tried to have a joint household account which Scott overdrew. She showed perseverance by removing her name from their joint account and keeping her retirement income in her own bank account. Shirley’s situation was unusual in that she and Scott had been married only 2 years at T1. Over time she made meaning about their situation, saying she was adjusting to the marriage as well as adjusting to his memory problems. For example, at T2 she said that she had been ready to “pitch him out” but his daughter would not take him in. By T3, she had reframed their problems in terms of marital adjustment, saying that his memory problems had not worsened and that her son had to help out financially at times. It is likely that if Scott’s cognitive problems noticeably increase, Shirley will once again be confronted with a need to further show her caregiving grit.

Although not every family dealt with financial issues (two caregivers did not mention money concerns), and it is likely a temporary problem for those who encountered it as the men with dementia eventually gave up control of household finances when their cognitive decline became undeniable, money management is an important aspect of dementia caregiving. Many wives struggled to balance their husband’s need to be a competent financial manager with ensuring their own financial well-being. Overspending in the short-term could have long-term implications for caregivers whose financial resources already may be stretched thin.

## Discussion

The experiences of 10 women providing long-term care for a PwD substantiate the process of adjusting to this type of caregiving and make important contributions to the literature in at least three domains. First, a longitudinal approach revealed that the women adjusted early on to the memory problems of their PwD, but their perseverance was challenged as their relative’s dementia progressed, and the PwD experienced personality and mood changes as well as a decline in physical health. The women did not necessarily make this connection, but it was evident from analyzing the transcripts that the practical problems of memory loss were managed (e.g., using notes and calendars), but the personality and social problems of the PwD became more pronounced over time, propelling the women into showing more grit by finding ways to persevere and remain committed over time.

Second, we applied the concept of grit to the study of dementia care as a novel means of furthering understanding of responses along the caregiving trajectory. Whether initially a caregiver is protective and does not want to confront the PwD about managing changes, or is more decisive and takes charge of the situation (Clemmensen, Busted, Søborg, & Bruun, 2019; Wawrziczny et al., 2016), both types of caregivers had to develop strategies over time to manage ongoing changes in their roles and relationships and cope with PwD’s cognitive and physical decline. As Duckworth (2016) noted, while some people seem to be innately gritty, everyone can strengthen perseverance and commitment and become grittier in order to cope with stressors. In the context of dementia caregiving, this means caregivers must continuously be realigning their sense of purpose (commitment) to the changes in caregiving tasks over time. These processes sustain their relationships and help caregivers maintain their own well-being by varying their strategies to cope with obstacles faced as the PwD’s health and cognitive abilities decline. Because our research was qualitative and longitudinal, evidence of grit emerged from the data analysis. Thus, we did not use the grit scale developed by Duckworth and Quinn (2009) to obtain a grit score for each participant. However, the grit scale is short, easy to administer (Duckworth & Quinn, 2009), and likely fruitful for future caregiving research.

Third, managing a partner’s identity may be an attempt by the women to delay the process of viewing themselves as caregivers, just as PwD resist identity change as their memory problems emerge (MacRae, 2011). In this sense, identity management is a coping strategy revealing perseverance and commitment to the relationship, or grit (Duckworth, 2016). In our study, the caregivers were active in the process of helping to maintain the PwD’s identity both in social settings and at home. Eventually, caregivers had to reevaluate their sense of purpose and come to terms with the reality of their situation and spend less energy preserving the PwD’s identity to allow more energy for managing day-to-day life with reduced practical and emotional support from their loved one.

A lost sense of intimacy was less salient with caregivers who experienced declines in physical function themselves (such as with Delia and Krystal), leading us to theorize that perceiving mutual age-related health problems could enable some spousal couples to maintain feelings of emotional closeness, particularly in the early stages of memory loss. That is, these couples may view themselves as growing old together and providing care to one another (Montgomery & Kosloski, 2013). For adult daughter caregivers who co-resided with their mothers and valued their emotionally close relationships, the loss of emotional intimacy was particularly difficult because they were losing not only a mother but one of their closest friends. Interestingly, only one of the caregivers (Gail, the adult daughter who was married) named another close relationship as a resource for managing the loss of emotional closeness with the PwD. However, all the women named work or serious hobbies as a source of coping and a place where they found purpose beyond their caregiving role and responsibilities.

### Limitations and Future Research

Although we found variation in how the women showed their grit over time, with some showing a lot of grit after assuming the caregiving role, but becoming more challenged in their commitment and perseverance as their care duties continued and intensified, we did not identify any caregivers showing no grit. It is possible that relatively short caregiving experiences do not elicit extensive grittiness, so caregivers with little or no grit did not participate in T4, or perhaps those having more difficulties did not remain in the study. Anyone can be gritty (Duckworth, 2016) and even those participants who were struggling with caregiving, among other stressors, demonstrated that they were able to develop more grit over time. Having uncovered evidence of grit in a long-term study of caregivers of PwD, we recommend continued research on commitment and perseverance in caregiving and the association of these aspects of grit with coping while caring for PwD.

A related area for future investigation stems from our small sample, which was not large enough to evaluate individual characteristics such as gender and racial differences or larger structural inequities including class, cultural, and geographic location in managing the challenges of long-term caregiving in the face of the stress that usually accompanies dementia care. However, other investigators have found meaningful differences that likely endure over time (Pearlin, Schieman, Fazio, & Meersman, 2005). Men tend to emphasize the instrumental support they provide care recipients and women emphasize emotional support as well as instrumental (Montgomery & Kosloski, 2013). African American caregivers of PwD spend nearly 10 hours more per week providing care than White caregivers do (Alzheimer’s Association, 2018), suggesting African American caregivers uphold familial values and cultural obligations regardless of personal costs (Potter, Roberto, Brossoie, & Blieszner, 2017; Stevens et al., 2004). Findings from the current study bring attention to the financial challenges and concerns caregivers face and the interconnected nature of gender and class power imbalances. Thus, researchers are encouraged to examine both individual and structural life course influences such as the differential effects of race, gender, class, and place on expressions of grit during caregiving. Understanding these influences would inform interventions designed to strengthen long-term caregivers’ ability to cope with changes in their roles and responsibilities over time.

### Translating Findings for Practice

Family members may expect to become caregivers but have few details about what caregiving for a PwD actually entails. As they begin their care journey, they want information about the progression of dementia, and occasionally professional help. However, they often resist embracing the new caregiving role, finding it difficult to accept relational shifts from a tie centered around mutually supportive interactions to one in which a partner is becoming increasingly dependent (Boots, Wolfs, Verhey, Kempen, & Vugt, 2015; Roberto, McCann, & Blieszner, 2013). Helping caregivers consciously develop more commitment to their relationship and more coping strategies to persevere in their caregiving role may be useful for enabling them to manage this shift. The caregivers we interviewed who showed the most grit took charge of their situations and consciously made decisions in the best interest of themselves and the PwD. Most people faced with new caregiving challenges want to do right by their relatives; encouraging them to view the transition to caregiving as a process they can control may have long-term positive benefits for families, particularly in coping with the PwD’s personality and mood changes. Consistent with Duckworth’s research (2016) and our findings, loved ones can learn to recognize that providing subtle social and emotional help is an early type of caregiving they can master, enabling them to develop adaptive coping strategies as new problems arise.

Moreover, caregivers may need help in understanding the progression of dementia and letting go of their caregiving role and responsibilities (Chen, 2016). Without this kind of support, caregivers may be reluctant to face their negative emotions and acknowledge the limits inherit in their situations, which can threaten their sense of purpose (i.e., Why am I doing this when it doesn’t help?). Caregivers also may need support in determining whether their primary goal is to provide good care or to have a close relationship with their spouse or parent (that might not be possible to sustain). Recognizing and accepting relationship changes could allow caregivers to share moments of intimacy with the PwD. We propose that acceptance of caring for a beloved but changed relative helps long-term caregivers to access and build grit as care demands increase.

Another way to help caregivers develop grit is to bring attention to potential financial problems early in the transition to caregiving. In addition to long-term financial planning, families should be encouraged to develop a system for day-to-day money management. Caregivers and PwD often recognize that cognitive functioning fluctuates with MCI, so having a plan in place—such as establishing two checking accounts to cover household expenses separately from discretionary spending—may enable better financial management across periods of cognitive fluctuation and more permanent decline.

Self-care should be framed as an area where caregivers can practice persistence, enhancing their grit. For example, having personal interests outside the relationship was a source of strength for the women we interviewed. In terms of grit and caregiving identity, pursuing personal interests for a few hours a week allowed the women time to reflect on and process the changes in their relationships with their PwD. Those new to caregiving should be encouraged to take advantage of resources that allow time for other activities, such as work or serious leisure pursuits, as making time for oneself is an important part of the transition to caregiving and management of care provision difficulties.

## Supplementary Material

igz121_suppl_Supplementary_Material
