# Supplementary material for: A Long and Winding Road: Dementia Caregiving With Grit and Grace
Source: Innov Aging. 2019 Aug 23;3(3):igz021. doi: 10.1093/geroni/igz021 (PMC6735788; doi:10.1093/geroni/igz021)
Supplement: igz121_suppl_Supplementary_Material [file igz121_suppl_supplementary_material.docx]

Appendix A

**T1 Interview Guide**

1. What changes did you notice in [name of person evaluated at the Memory Clinic] that led to [her/his] visit to the Memory Clinic? When did these changes start? How long have you been dealing with minor and/or major difficulties with [name’s] memory?
2. Did you go anywhere else before contacting the Memory Clinic? What happened? What was your reaction to that?
3. What is your reaction to the test results you got from the Memory Clinic? What is [name’s] reaction? How about the rest of the family? Is there anyone who could be involved in dealing with this situation and is not?
4. I know that you are [name’s] [wife/husband, daughter/son, friend, etc.], but besides that, what do you see as your role and relationship in helping [her/him] deal with the changes that are happening? How do you feel about that?
5. What are you doing to manage everyday life with [name]? Are you getting any outside help now that is different from help you got before? What kind? From whom?
6. Has [name’s] doctor given you any information or suggestions for dealing with this situation? Have you found any helpful information elsewhere about memory loss in later life? What kind? Where did you get it? Is any of this helpful? In what ways?
7. Are you taking care of anyone else these days [child, parent, friend, etc.]? Do you get any help with that? From whom? Is that caregiving going to continue in the future?
8. What about your own physical and mental health these days—have you noticed any changes since [name’s] situation began? What are you doing to make sure you stay as healthy as possible? Have you had to lessen or give up anything you found enjoyable before?
9. What do you think will happen with [name] in the future in terms of thinking and memory changes?
10. What kinds of information and assistance do you think [name] and you might need in the future? How comfortable would you feel about asking family members or friends for help? What about community agencies or senior services—do you know where to turn for help from them? Would you be willing to rely on them?
11. You might be faced with making some important decisions about [name] in the future [e.g., living arrangements, help with daily matters]. How have you gone about determining what to do in the past if an important decision [about anything] came up? Who will be involved in making decisions about future plans for [name]? Is that different from before?
12. What suggestions would you give others about how to make living with memory loss easier?
